# Modulating Reconsolidation With Non-invasive Brain Stimulation—Where We Stand and Future Directions

**DOI:** 10.3389/fpsyg.2018.01430

**Published:** 2018-08-13

**Authors:** Marco Sandrini, Antonio Caronni, Massimo Corbo

**Affiliations:** ^1^Department of Neurorehabilitation Sciences, Casa Cura Policlinico, Milan, Italy; ^2^Department of Psychology, University of Roehampton, London, United Kingdom

**Keywords:** reconsolidation, reactivation, retrieval, memory, tDCS, TMS, brain stimulation, patients

This opinion article highlights the importance of targeting critical brain regions during reconsolidation to gain insight into the brain mechanisms of memory dynamics and modulating existing memories.

Accumulating evidence has shown that reactivated existing memories become sensitive to modification during reconsolidation (Sandrini et al., 2015; Lee et al., 2017; see Figure 1). This post-reactivation state of plasticity is a topic of intense scientific investigation not only for the basic understanding of memory processes but also for the development of novel clinical interventions to modulate existing memories.

**Figure 1:**
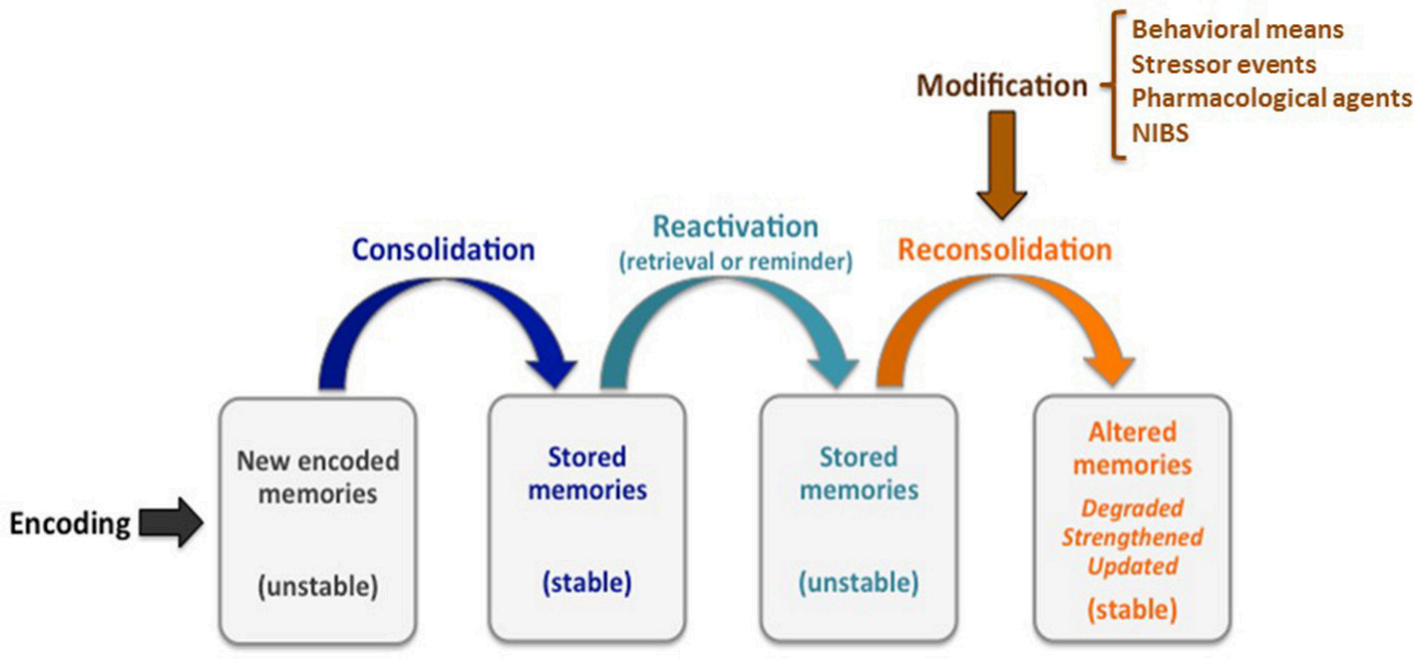
For a limited time after encoding, memories undergo an initial fragile/unstable phase, before being stabilized through the consolidation process. However, consolidated memories may return to a fragile phase when they are retrieved or reactivated by a reminder, thus requiring a restabilization process that is known as reconsolidation. During this time-limited reconsolidation window, existing memories can be degraded, strengthened, or updated by the inclusion of new information. Memories can be modified through behavioral means (e.g., interference, extinction), stressor events, pharmacological agents (e.g., propranolol, a beta blocker) or noninvasive brain stimulation (NIBS) techniques. Modified from Sandrini et al. (2015) with permission from Elsevier.

Noninvasive brain stimulation (NIBS) (Dayan et al., 2013) is a safe approach for studying brain mechanisms of memory reconsolidation. A seminal repetitive transcranial magnetic stimulation (rTMS) study showed that the primary motor cortex (M1) is essential for successful modification of motor memory strength (Censor et al., 2010). Subsequent rTMS work demonstrated that lateral prefrontal cortex (PFC) plays a causal role in strengthening episodic memory through reconsolidation (Sandrini et al., 2013). Similar effects on episodic memory have been documented with transcranial direct current stimulation (tDCS) applied to the PFC in young and older adults (Javadi and Cheng, 2013; Sandrini et al., 2014; Manenti et al., 2017). In addition, tDCS to the PFC after a reminder induced longer-lasting positive effects (up to 1 month) relative to tDCS during encoding (Manenti et al., 2016). Future NIBS work should investigate whether beneficial effects can be observed also in individuals with Mild Cognitive Impairment (MCI)—a population at risk of developing dementia such as Alzheimer's Disease (AD).

Regarding the possibility to disrupt “intrusive” maladaptive memories, a pilot study showed that the combination of brief exposure to a traumatic event with deep rTMS to the medial PFC induced beneficial effects in patients with post-traumatic stress disorder (Isserles et al., 2013). Another study showed that tDCS applied to the PFC after a reminder enhanced fear memories (Mungee et al., 2014). However, there is a lack of evidence that NIBS can disrupt maladaptive memories through reconsolidation. So far, only electroconvulsive therapy administered after memory reactivation in patients with unipolar depression has been shown to disrupt reactivated, but not non-reactivated, emotional episodic memories (Kroes et al., 2014).

Overall, it is important to keep in mind that the observed behavioral effects, up to now, are very transient and modest, despite encouraging. Replication studies from independent research groups are also needed.

Future research should also use rhythmic rTMS or transcranial alternating current stimulation to study the causal role of neural oscillations (e.g., in the beta frequency, Hanslmayr et al., 2014) for memory reconsolidation. In addition, the combination of NIBS with imaging offers the possibility to identify the causal systems-level mechanisms underlying memory reconsolidation (Censor et al., 2014a,b). For example, M1-rTMS interference with reactivated motor memory modulated M1-striatum intrinsic functional connectivity, which predicted offline memory modification (Censor et al., 2014b).

From a clinical perspective, an important issue that requires investigations is the interaction between NIBS during reconsolidation and pharmacological interventions. Several neurotransmitter systems (e.g., dopaminergic, serotonergic, cholinergic, and noradrenergic) have a role in modulating plasticity (Nitsche et al., 2012), and there is evidence that medications from very different pharmacological classes can enhance or hinder both NIBS-induced excitatory and inhibitory plasticity [i.e., long-term potentiation (LTP) and long-term depression (LTD)-like plasticity, respectively]. Effects of central nervous system (CNS) agents on NIBS-induced plasticity are complex and depend on different factors among which drug type and dosage, disease stage, NIBS type, and protocol. Lorazepam, a widely used benzodiazepine, delays, enhances, and prolongs LTP-like plasticity elicited by tDCS, but has no effect on tDCS-induced LTD-like plasticity (Nitsche et al., 2004a,b). The widely prescribed citalopram, a serotonin reuptake inhibitor antidepressant, enhances, and prolongs tDCS induced LTP-like plasticity, while reverting tDCS induced LTD-like plasticity to LTP-like plasticity (Nitsche et al., 2009). Among psychoactive substances, nicotine (Thirugnanasambandam et al., 2011) and alcohol (Conte et al., 2008) both cause complex modulation of NIBS-induced plasticity. It has been pointed out that studies on the clinical effectiveness of NIBS-induced plasticity in patients should take into account the concomitant use of multiple medications and the importance of reporting medication use in NIBS clinical trials (Huang et al., 2017; McLaren et al., 2018). In this regard, it is noteworthy that several non-CNS agents can actually modulate NIBS-induced plasticity. For example, antihypertensive drugs (e.g., beta blockers) reduce both tDCS LTP and LTD-like plasticity (Nitsche et al., 2004a). Alpha blockers, often used for treating urinary symptoms, abolish LTP-like plasticity induced by paired associative stimulation (PAS) (Korchounov and Ziemann, 2011). In real world context, patients that would benefit from NIBS-induced plasticity are often elderly people with comorbid conditions who are chronically taking several medications. As an example, sedatives, antidepressants, and antihypertensives are common medications for stroke patients—a condition in which NIBS-induced plasticity is often studied as a therapeutic tool. In view of the above, unchecked medications could cause unwanted, detrimental interactions with NIBS-induced plasticity. However, we want to stress that there is a strong rationale according to which NIBS and medications can actually work together for boosting therapeutic plasticity, eventually leading to a better clinical outcome (Perez et al., 2014). For example, the combination of tDCS and antidepressant drugs increases the efficacy of each single treatment in major depression (Brunoni et al., 2013) and tDCS associated with citalopram enhances declarative memory formation in young and older adults (Prehn et al., 2017).

Since NIBS techniques are promising interventions for treating declarative memory impairments that are often the first symptoms of dementia, the combination of NIBS with pharmacological interventions could prove to be an important symptomatic therapy for subjects with MCI due to AD (Albert et al., 2011), which today represents the population of interest for the use of the so-called Disease-Modifying Therapies (DMTs).

Finally, future research should address the issue of inter-individual variability in the effects of NIBS during reconsolidation. In particular, the possibility that variations in the presence of specific genetic polymorphisms (e.g., brain-derived neurotrophic factor gene; Chaieb et al., 2014) or brain structure (i.e., gray and white matter integrity; Censor et al., 2016) may influence the individual responsiveness are still open questions.

In conclusion, a better understanding of these basic and clinical issues will be instrumental to the development of NIBS clinical interventions for improving memory and symptoms through reconsolidation.

## Author contributions

All authors listed have made a substantial, direct and intellectual contribution to the work, and approved it for publication.

### Conflict of interest statement

The authors declare that the research was conducted in the absence of any commercial or financial relationships that could be construed as a potential conflict of interest.
